# Predictors of sexual violence among female students in higher education institutions in Ethiopia: A systematic review and meta-analysis

**DOI:** 10.1371/journal.pone.0247386

**Published:** 2021-02-19

**Authors:** Bereket Kefale, Melaku Yalew, Yitayish Damtie, Mastewal Arefaynie, Bezawit Adane

**Affiliations:** 1 Department of Reproductive and Family Health, School of Public Health, College of Medicine and Health Sciences, Wollo University, Dessie, Ethiopia; 2 Department of Epidemiology and Biostatistics, School of Public Health, College of Medicine and Health Sciences, Wollo University, Dessie, Ethiopia; University of Michigan, UNITED STATES

## Abstract

**Background:**

Sexual violence is a profound social and public health problem in Ethiopia. Female students in institutions of higher education are highly vulnerable to sexual violence. Different studies conducted on sexual violence at higher education institutions lack consistency and inclusiveness. Thus, this systematic review and meta-analysis were conducted to estimate the lifetime and twelve-month prevalence, and predictors of sexual violence among female students in institutions of higher education in Ethiopia.

**Methods:**

This study used a systematic review and meta-analysis of studies conducted from January 1, 2000, to June 1, 2020, in Ethiopia. This review followed Preferred Reporting Items for Systematic Reviews and Meta-Analyses (PRISMA) guidelines. Electronic databases including PubMed, Cochrane Library, Hinari, Google Scholar, CINAHL, and Global Health were searched using relevant search terms. Meta-analysis was performed using STATA 14 software. The I^2^ statistics and Egger’s test were used to assess heterogeneity and publication bias, respectively. Forest plots were used to present the prevalence and odds ratio (OR) with a 95% confidence interval (CI).

**Results:**

This systematic review and meta-analysis included 10 studies, 5790 study participants. The pooled lifetime and twelve-month prevalence of sexual violence among female students in Ethiopia was 49.4% (95%CI: 37.87, 60.96) and 36.02% (95%CI: 26.42, 45.62) respectively. Rural residence (OR = 2.13;95%CI: 1.33, 3.42), alcohol drinking (OR = 2.03; 95%CI: 1.44, 2.87), and ever had a boyfriend (OR = 2.07; 95%CI: 1.32, 3.62) were factors associated with sexual violence.

**Conclusions:**

The lifetime prevalence of sexual violence among female students in institutions of higher education in Ethiopia was high. Place of residence, alcohol drinking, and ever had a boyfriend were statistically significant factors of sexual violence. Life skill training and law enforcement are needed to control alcohol consumption. Additionally, more focused interventions should be done in rural settings.

**Registration:**

This systematic review has been registered in the International Prospective Registry of Systematic Review (PROSPERO) with a specific registration number CRD42020155894.

## Introduction

Violence against women is a significant public health problem, as well as a fundamental violation of women’s human rights [1]. Due to its nature, the magnitude of violence against women is frequently “hidden” resulting in a significant underestimation. Globally, about 35% of women have experienced either physical and/or sexual intimate partner violence or non-partner sexual violence [2]. A Systematic Review and Meta-Analysis in Sub-Saharan Africa showed that 26.2% of female youths in educational institutions were victimized by sexual violence [3]. Ethiopian Demographic and Health Survey (EDHS) 2016 reported that 10% of women aged 15–49 in Ethiopia have experienced sexual violence [4].

University students are a great potential resource for a country. However, substantial proportions of female students in institutions of higher education in Ethiopia have been subjected to sexual violence and its consequences due to different reasons [5–7]. Some of these reasons include lack of family control, the need to explore their newly discovered freedom, presence of pimp and pubs surrounding the campus, sexual experimentation, peer pressure, weak institutional administration, lack of comprehensive knowledge on sexual and reproductive health problems, substance use and financial insecurity [8–10].

Various studies revealed that older age [11], age at first sex [12], level of education [5, 6], rural residence [5, 11, 13–15], alcohol drinking [5, 11–13, 16], lack of pocket money [16], witnessed maternal violence [5, 11, 13], ever had a boyfriend [6, 14], ever had sex [17], unmarried parental status [5, 16, 17], non-discussion of sexual issues with family [11, 15] and low level of maternal educational status [7] are factors associated with sexual violence.

Sexual violence has serious short-term and long-term physical, mental, sexual, and reproductive health problems for victims. These include; Human immune-deficiency virus (HIV) and other sexually transmitted infections [18–20], induced abortion [21–23], harmful alcohol use [24, 25], school dropout [26, 27], depression, anxiety, and suicide [28–33], and other fatal and non-fatal injuries [34–37].

Elimination of all forms of violence against women and girls is one of the key Sustainable Development Goals (SDG), target 5.2 [38]. It is also crucial to achieve most of the SDGs particularly Goal 3 and Goal5. Due to this, the prevention of violence against women has long been the focus of attention by several global and national organizations. The government of Ethiopia has also adopted, ratified, and revised various legislation and policies to fight violence against women [39–41]. However, sexual violence has continued to be a major public health problem in Ethiopia.

Determining the magnitude of sexual violence among University students is crucial for designing and implementing effective interventions to reduce the problem. However, the studies conducted in Ethiopia found inconsistent and inconclusive findings regarding the prevalence and its predictors. The lifetime prevalence of sexual violence among female students in institutions of higher education in Ethiopia was varied from 14.3% in Hawassa University [14] to 76.4% in Ambo University [5]. Moreover, the studies showed inconsistent findings regarding factors associated with sexual violence [5–7, 11–17]. Therefore, this review used the evidence of these studies to estimate the pooled lifetime and twelve-month prevalence, and predictors of sexual violence among female students in institutions of higher education in Ethiopia.

## Methods

### Registration

This systematic review and meta-analysis strictly followed the Preferred Reporting Items for Systematic Review and Meta-Analysis (PRISMA) guidelines [42] (S1 File). It has been registered in the International Prospective Registry of Systematic Review (PROSPERO) with a specific registration number CRD42020155894.

### Study design and search strategy

A systematic review and meta-analysis of published and unpublished studies were conducted to assess the pooled prevalence and associated factors of sexual violence among higher education institutions in Ethiopia. The databases used to search for studies were: PubMed, Cochrane Library, Hinari, Google Scholar, CINAHL, and Global Health. The search was made using the following key terms: "prevalence", "magnitude", "proportion", "incidence", "Sexual violence", "sexual abuse", "sexual coercion", "sexual harassment", "sexual assault", "rape", "violence", "gender-based violence", "assault", "factors", "determinants", "predictors", "factors associated", "associated factors", "risk factors", "University", "College", "Higher education institutions", "campus", "students", "undergraduate students", "female", "Ethiopia". All key terms were searched by a combination of Boolean operators “AND” or “OR” as appropriate and the search was done by two authors independently (BK and YD).

### Inclusion and exclusion criteria

All available studies conducted from January 1, 2000, to June 1, 2020, were included in this review. This review included published and unpublished studies that were conducted on the prevalence of sexual violence and its determinants in the higher education institutions in Ethiopia. All observational studies with English language publication which measured the prevalence of sexual violence; sexual coercion, sexual harassment, and rape among female students were included in this review. However, pure qualitative studies, studies with poor methodological quality, and those not reporting the outcome of interest were excluded from the review.

### Definition of sexual violence

The World Health Organization (WHO) defines sexual violence as “any sexual act, attempt to obtain a sexual act, unwanted sexual comments or advances, or acts to traffic or otherwise directed against a person’s sexuality using coercion, by any person regardless of their relationship to the victim, in any setting, including but not limited to home and work” [43].

### Study selection, quality appraisal, and data extraction

All articles identified from selected databases were exported to Endnote X8 and duplicate files were excluded. The remaining articles and abstracts were independently screened by two groups (YD and MA) for inclusion in the full-text appraisal. The difference between reviewers was managed through discussion and disagreement was handled by the third party (BA). The quality of articles that met inclusion criteria were assessed using the Joanna Briggs Institute (JBI) critical appraisal checklist [44]. The tool has a separate appraisal checklist for each type of study design. Two reviewers independently assessed articles before inclusion for review. Articles with quality scores of fifty and above were included in the final review.

Data were extracted using Microsoft excel 2010 sheet. The data extraction tool contains information on the author’s name, year of study, year of publication, study setting (university or college students), response rate, sample size, outcome measured, prevalence, adjusted odds ratio (AOR), and upper and lower confidence interval (CI) of AORs.

### Statistical methods and analysis

The analysis was conducted using STATA 14 software. Forest plots were used to show the lifetime and twelve-month (current) prevalence of sexual violence among female students in higher education institutions of Ethiopia. Due to the presence of substantial heterogeneity among studies, the random effect model of analysis was used. The heterogeneity test of included studies was assessed by using the I^2^ statistics, and it was declared using a p-value of less than 0.05 [45]. Subgroup analyses were also conducted by different study characteristics such as study setting (university or college), region (Amhara, Oromia, southern nations, nationalities, and people’s region (SNNRR)) and study year (before 2015 or 2015 and above). The different factors associated with sexual violence were presented using odds ratios (ORs) with 95% CI. The Egger regression asymmetry test was used to assess publication bias [46, 47]. The presence of publication bias was declared with a p-value of less than 0.05.

## Results

### Study selection

**Fig 1:**
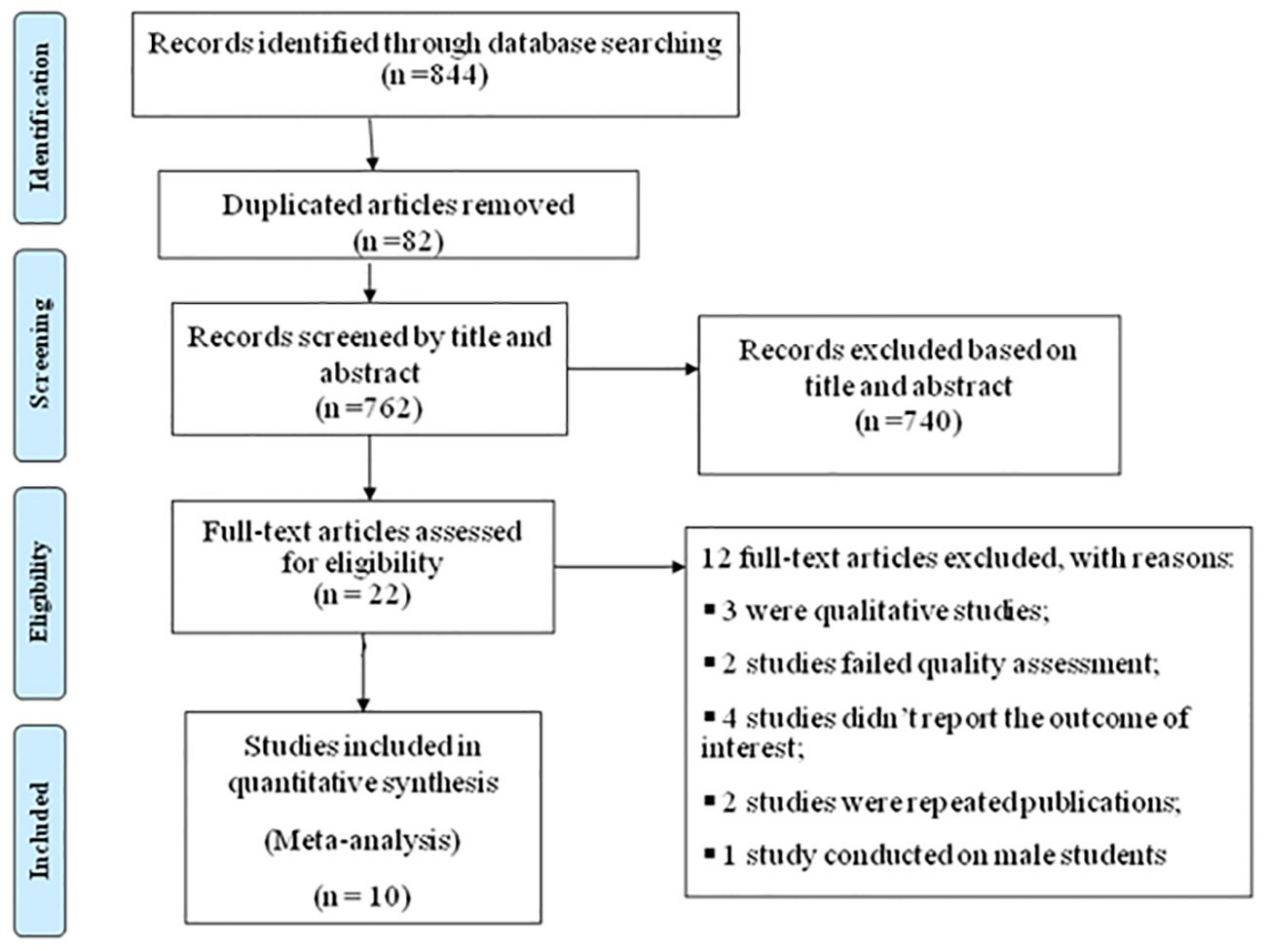
PRISMA flow diagram of the included studies for the systematic review and meta-analysis of sexual violence among female students at a higher education institution in Ethiopia.

This systematic review and meta-analysis included both published and unpublished studies conducted on sexual violence among female students at higher education institutions in Ethiopia. A total of 844 records were retrieved through electronic database searching. From these, 82 duplicated records were excluded, and the remaining 740 articles were excluded using their titles and abstracts. Twenty-two full-text articles were assessed for eligibility. From these, 12 full-text articles were excluded for prior criteria, and a total of 10 studies were included in the review (Fig 1).

### Characteristics of included studies

All articles included in this review were cross-sectional studies [5–7, 11–17]. The sample size of studies ranged from a minimum of 336, a study conducted at Hawassa University [14] to a maximum of 1330, another study conducted in colleges of Hawassa city [13]. Overall, this review included a total of 5790 study participants. The studies were conducted from 2006 to2018 in different regions of the country (Table 1).

**Table 1:** Summary characteristics of studies included in the systematic review and meta-analysis.

Authors and year	Study area	Study design	Sample size	Response rate	Outcome measured	Prevalence	Quality score
Henok A etal, 2015	Mizan-Tepi University	Institutional based cross-sectional	570	94.4	Sexual harassment	66.3	78%
Bekele and Deressa, 2014	Ambo University	Institutional based cross-sectional	590	98.8	Sexual coercion	76.4	83%
Takele and Setegn, 2014	Madawalabu University	Institutional based cross-sectional	397	96.6	Sexual violence	41.1	78%
Arnold D et al, 2008	Hawassa city	Institutional based cross-sectional	1330	100	Sexual violence	46.3	56%
Shimekaw B etal, 2013	Bahir Dar city	Institutional based cross-sectional	536	99.1	Sexual violence	37.3	67%
Adinew and Hagos, 2017	Wolaita Sodo University	Institutional based cross-sectional	462	95.4	Sexual violence	45.5	72%
Benti and Teferi, 2015	Nekemet town	Institutional based cross-sectional	562	99.6	Sexual coercion	41.3	67%
Kassa S etal, 2019	Wollo University	Institutional based cross-sectional	402	97.8	sexual coercion	59.7	72%
Sendo and Meleku, 2015	Hawassa University	Institutional based cross-sectional	336	100	Rape	14.3	61%
Bekele T etal, 2015	Madawalabu University	Institutional based cross-sectional	605	97.9	Sexual harassment	66.0	72%

### Prevalence of sexual violence

**Fig 2:**
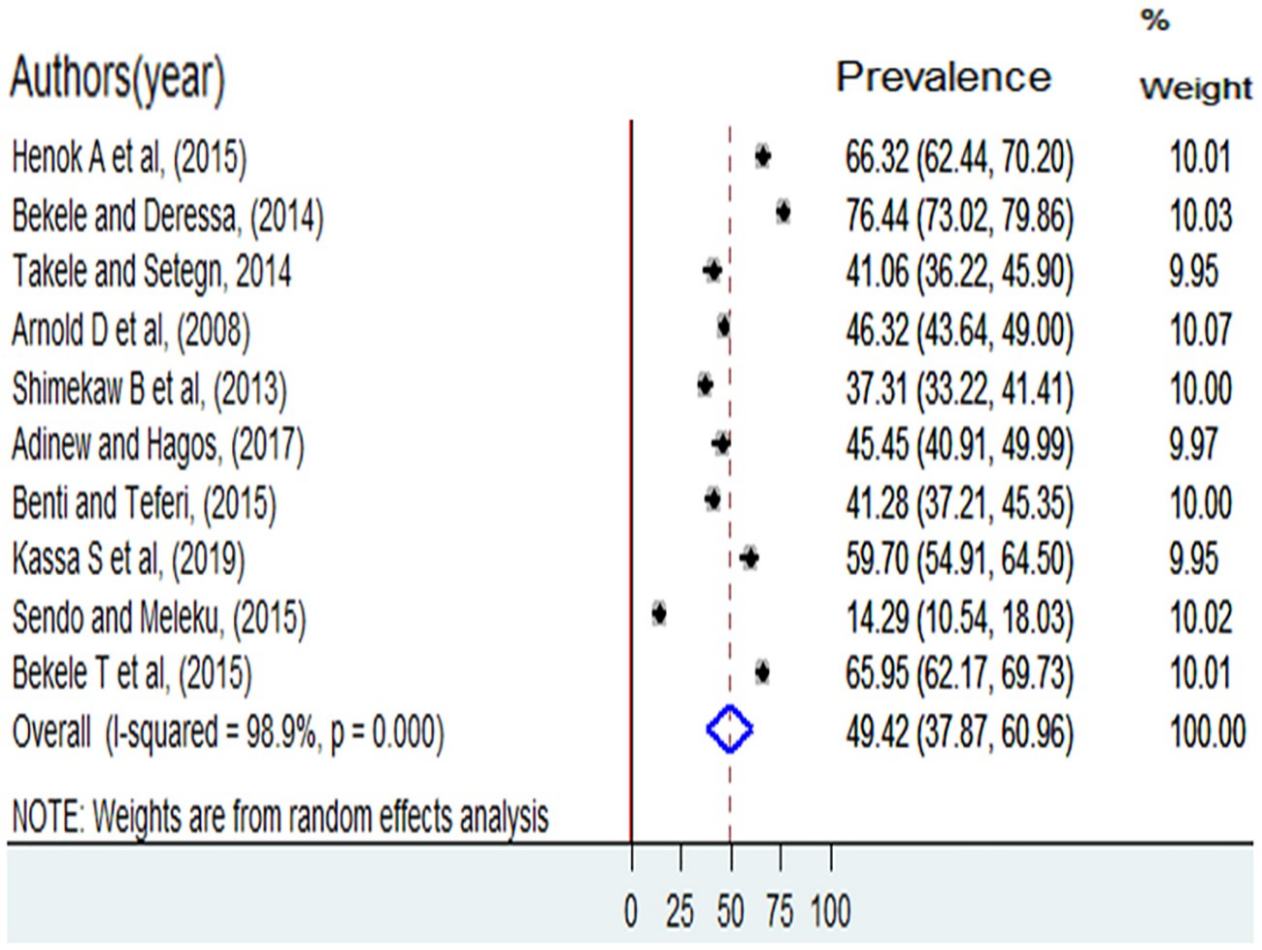
The lifetime prevalence of sexual violence among female students in higher education institution in Ethiopia, 2006 to 2018.

**Fig 3:**
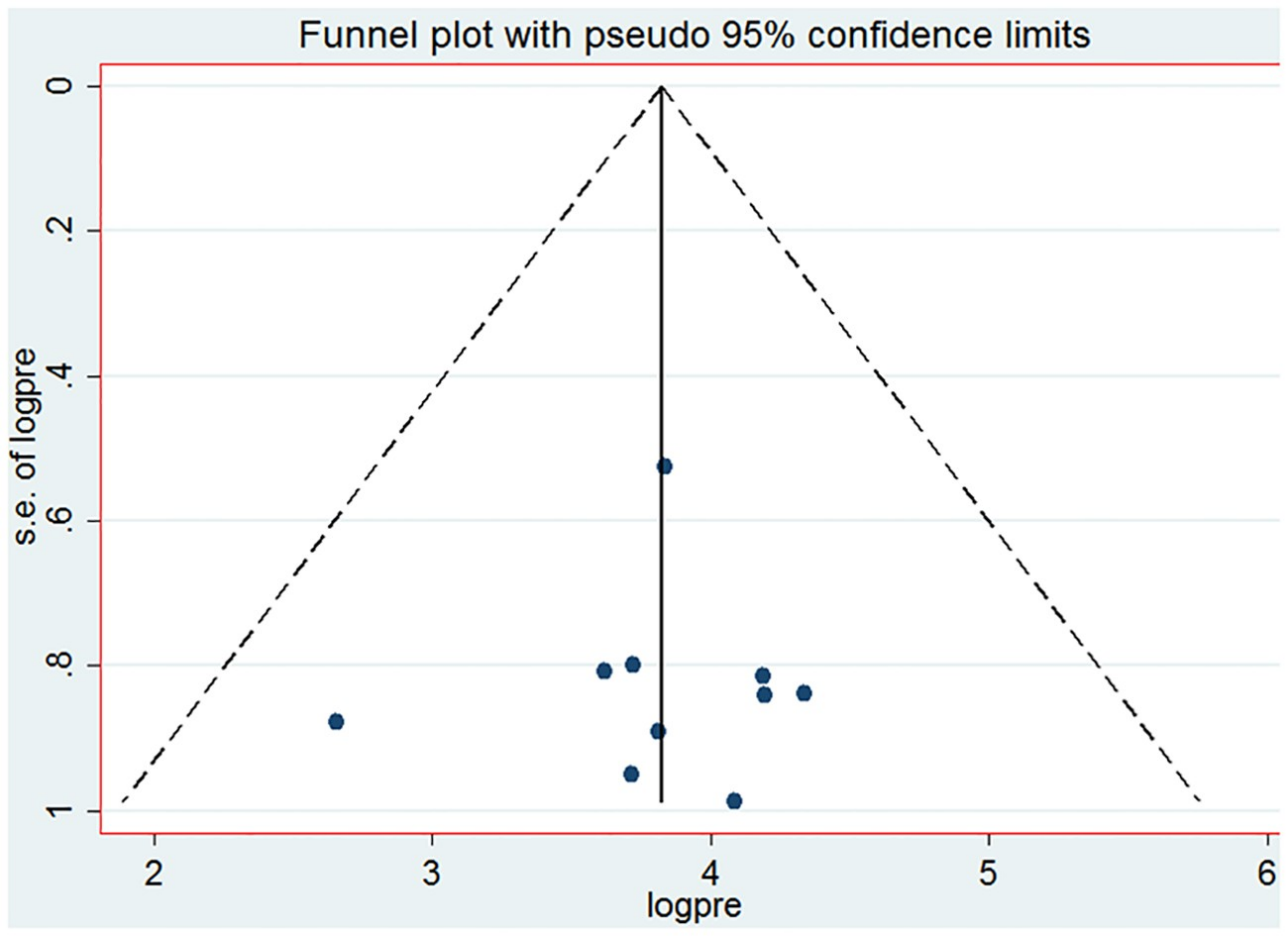
Funnel plot with 95% confidence limit of pooled lifetime prevalence of sexual violence among female students in higher education institution of Ethiopia, 2006 to 2018.

The overall pooled lifetime prevalence of sexual violence among females in higher education institutions in Ethiopia was 49.4% (95%CI: 37.87, 60.96). The highest lifetime prevalence of sexual violence was reported from a study done at Ambo University. The study showed that 76.4% of female students had experienced sexual violence [5]. The lowest prevalence of sexual violence was 14.3% among female students at Hawassa University [14]. Significant heterogeneity was observed among included studies in the meta-analysis, I^2^ = 98.9%, p < 0.001 (Fig 2). The funnel plot showed a symmetrical appearance (Fig 3). The Egger’s regression asymmetry test also showed non-significant publication bias, p-value = 0.61.

**Fig 4:**
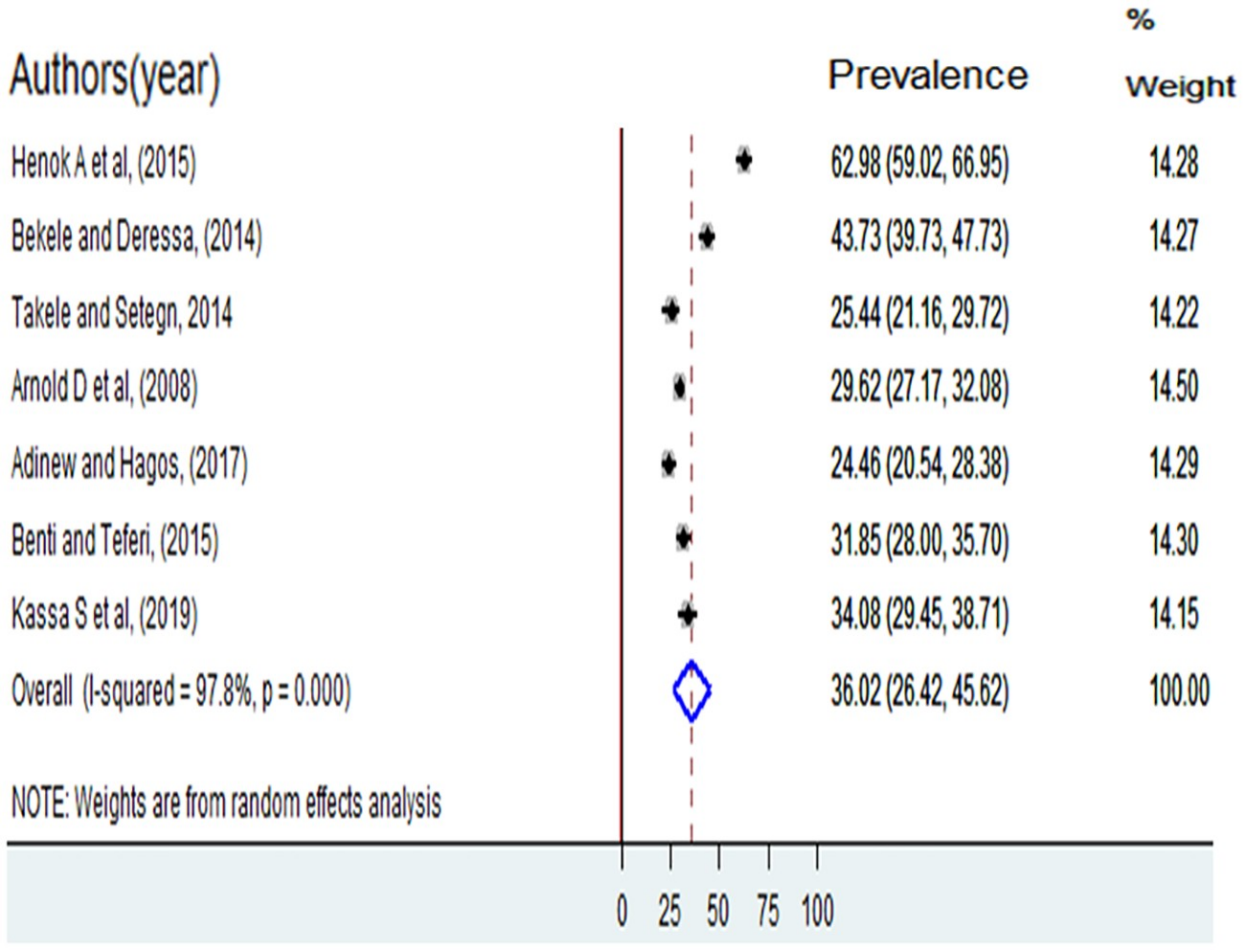
Twelve-month prevalence of sexual violence among female students at a higher education institution in Ethiopia, 2006 to 2018.

**Fig 5:**
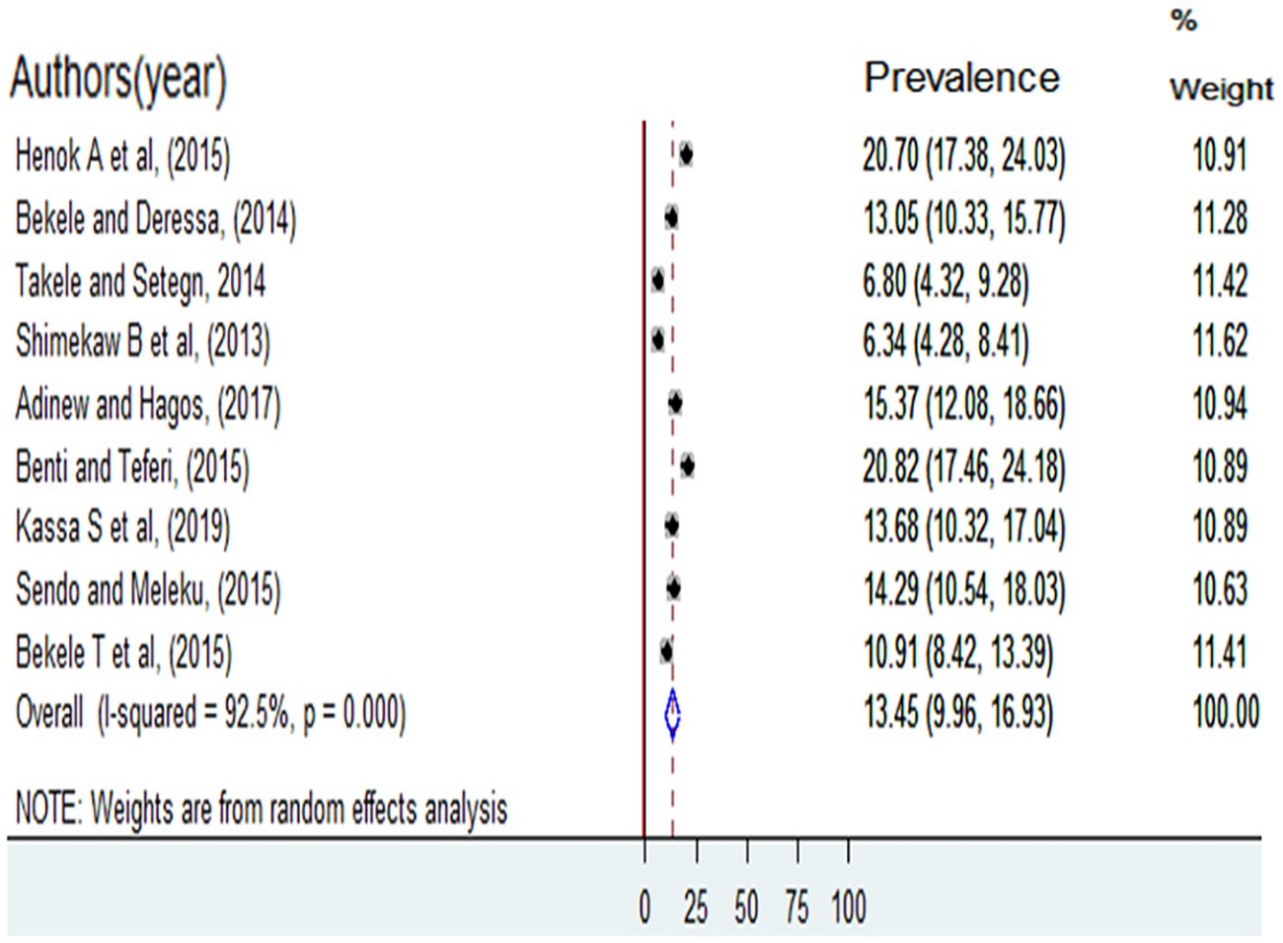
Prevalence of completed rape among female students at higher education institution of Ethiopia, 2012 to 2018.

The twelve-month (current) prevalence of sexual violence was reported by seven studies [5, 6, 11–13, 16, 17] and it ranged from 24.46% among female students in Wolaita Sodo University [11] to 63.98% among female students in Ambo University [5] and the pooled twelve-month prevalence of sexual violence was36.02% (95%CI: 26.42, 45.62) (Fig 4). A total of nine studies [5–7, 11, 12, 14–17] were reported prevalence of completed rape. The highest prevalence was 20.82% among college female students in Nekemet, and the lowest prevalence was 6.34% among college female students in Bahir Dar. The pooled prevalence of completed rape was 13.45 (95%CI: 9.96, 16.93) (Fig 5).

### Sub-group analysis

Subgroup analyses were also conducted by different study characteristics such as study setting, region, and study year to identify the source of heterogeneity however, the heterogeneity still exists. Thus, the heterogeneity may be explained by other factors not included in this review. The lifetime prevalence of sexual violence among college students and university students was 52.75 (95%CI: 35.90, 69.61) and 41.81 (95%CI: 36.34, 47.28) respectively. The lifetime prevalence of sexual violence in the Oromia region was 56.23 (95%CI: 38.53, 73.92) (Table 2).

**Table 2:** Subgroup analysis of the prevalence of sexual violence at higher education institutions in Ethiopia, 2006–2018.

Subgroup	Number of studies	Total sample	Prevalence (95%CI)	Heterogeneity	
				I^2^	p-value
By study setting					
University	7	3362	52.75 (35.90, 69.61)	99.2	< 0.001
College	3	2428	41.81 (36.34, 47.28)	85.7	0.001
By region					
Amhara	2	938	48.47 (26.53, 70.41)	97.9	< 0.001
Oromia	4	2154	56.23 (38.53, 73.92)	98.7	< 0.001
SNNPR	4	2698	43.09 (22.96, 63.22)	99.2	< 0.001
By study year					
Before 2015	8	4926	48.63 (34.73, 62.54)	99.1	< 0.001
2015 and above	2	864	52.56 (38.59, 66.52)	94.4	< 0.001
Total	10	5790	49.42 (37.87, 60.96)	98.9	< 0.001

SNNPR- southern nations, nationalities, and people’s region.

### Predictors of sexual violence

**Fig 6:**
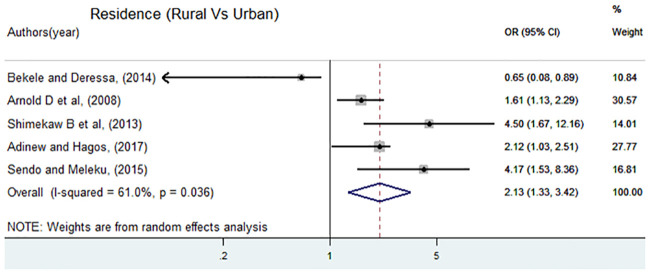
Forest plot of odds ratio for the association of residence and sexual violence in Ethiopia.

There were five predictors frequently reported by the studies included in the review. These predictors were place of residence, alcohol drinking, ever had a boyfriend, ever witnessed maternal violence, and having multiple sexual partners. Out of these predictors, ever witnessed maternal violence had a positive significant association in all studies [5, 7, 11, 13, 17] reported it. Thus, the factors included in this analysis were the place of residence, alcohol drinking, ever had a boyfriend, and having multiple sexual partners. A separate analysis was conducted for each variable. A total of five articles [5, 11, 13–15] were included to determine the association between place of residence and sexual violence. Four of the included studies found a positive significant association, the rest showed a negative association. The final pooled meta-analysis showed that the odds of experiencing sexual violence among female students who resided in rural areas were 2.1 times higher than female students who lived in urban areas, OR = 2.13 (95%CI: 1.33, 3.42) (Fig 6).

**Fig 7:**
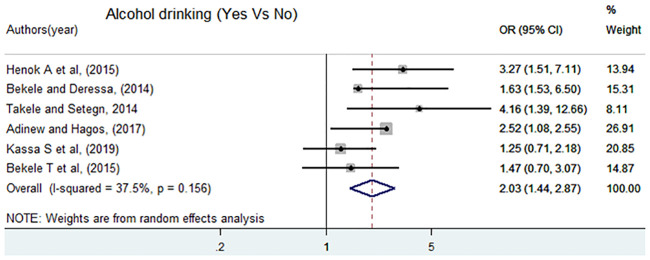
Forest plot of odds ratio for the association of alcohol drinking and sexual violence in Ethiopia.

A total of six articles [5–7, 11, 12, 17] were included to assess the effect of alcohol drinking and sexual violence. Four of the included studies had a positive significant association and the other two studies showed a non-significant association. The pooled meta-analysis showed that the odds of experiencing sexual violence among female students who drank alcohol were two times higher than female students who were never drinking alcohol, OR = 2.03 (95%CI: 1.44, 2.87) (Fig 7).

**Fig 8:**
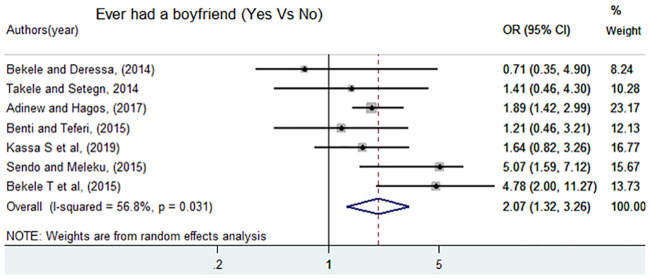
Forest plot of odds ratio for the association of ever had a boyfriend and sexual violence in Ethiopia.

Similarly, seven studies [5, 7, 11, 12, 14, 16, 17] were included to assess the association of ever had a boyfriend and sexual violence. From these, three studies had a positive significant association with sexual violence, and four studies showed a non-significant association. However, the pooled meta-analysis showed that female students who ever had boyfriend had higher odds of experiencing sexual violence than female students who do not have boyfriends, OR = 2.07 (95%CI: 1.32, 3.62) (Fig 8).

**Fig 9:**
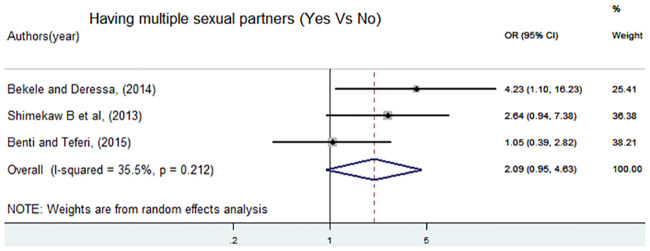
Forest plot of odds ratio for the association of having multiple sexual partner and sexual violence in Ethiopia.

Additionally, three articles [5, 15, 16] were included to assess the association of having multiple sexual partner and sexual violence. Out of these, two studies had a non significant association with sexual violence, and the rest showed a positive significant association. The pooled meta-analysis also showed a non significant association OR = 2.09 (95%CI: 0.95, 4.63) (Fig 9).

## Discussion

This systematic review and meta-analysis were conducted to estimate the prevalence and determinants of sexual violence among female students at higher education institutions in Ethiopia. The pooled lifetime prevalence of sexual violence among higher education institution female students in Ethiopia is 49.4% (95%CI: 37.87, 60.96). This finding is much higher as compared to 26.22% in Sub-Saharan Africa [3], 15.3% in China [48], and 15.0%-19.2% among reviews conducted worldwide [49–51]. This might be due to a difference in the study population. In this review, the study populations are university and college students. However, study populations in the review conducted in Sub-Saharan Africa were secondary school, and college and university students. The study populations among reviews conducted in China and the world were also adolescents less than 18 years old. The other possible reason might be there is weak law enforcement and gender in equality in Ethiopia compared with China and other countries included in the review [52]. The finding of this review shows how much it is too far to achieve global and national commitment to end violence against women [1, 38, 39]. Thus, it urges the government and other concerned bodies to invest their effort to address the problem of sexual violence at higher education institutions.

The lifetime prevalence of sexual violence among university female students was higher than college students. This might be due to university students live in the campus with full independence without family control, but most of the college students live with their family or a town near to their family with close monitoring and support. Furthermore, the presence of night clubs, pubs, pimps, and other substance-related business houses surrounding the universities make female students highly vulnerable to sexual violence compared to female students in the colleges [8].

Female students who reside in rural areas were more likely to experience sexual violence than those who live in urban areas. This finding is similar to a review in Sub-Saharan Africa [3]. This might be due to rampant harmful beliefs and traditions, inadequate legal provisions, inaccessibility of legal services, unequal power relationships, and low level of awareness on sexual violence in rural areas [53–55]. Female students from the rural areas may also live far from their family in the dormitory which increases the possibility of being victimized by sexual violence.

The pooled odds of experiencing sexual violence among female students who drink alcohol were higher than female students who never drink alcohol. Other systematic reviews also found similar findings [3, 25, 56]. The reason for this could be alcohol use can alter the level of consciousness and problem-solving skill. Alcohol also increases one’s willingness to take risks, making individuals less aware of and concerned by the consequences of their behavior [57–59].

Female students who ever had boyfriend had higher odds of experiencing sexual violence than female students who did not have boyfriends. Females who had boyfriend trust their friend and spent much time with them in more risky places such as night clubs, couple houses, and substance houses that make them vulnerable to sexual violence. As evidenced by different studies sexual violence is most frequently perpetrated by intimate partners (boyfriends or husbands) [60]. Moreover, those students whoever had a boyfriend may be influenced by peers to be victimized by sexual violence.

The PRISMA guideline was strictly followed throughout all steps of the systematic review and meta-analysis. However, all articles included in this review were cross-sectional, which limits the causality of predictors on sexual violence. Since variables associated with sexual violence were reported in a few studies; this review assessed only three factors affecting sexual violence. However, sexual violence could be affected by many factors such as behavioral, environmental, institutional, socioeconomic, and cultural factors.

## Conclusions

Almost half of the female students had experienced sexual violence in Ethiopia. Being rural residents, alcohol drinking, and ever had a boy friend were found to be associated with sexual violence. The governments and relevant stakeholders should develop and implement effective educational institution based interventions to address sexual violence against female students in higher education institutions. Implementation of laws that control alcohol consumption is also crucial. Moreover, the institutions of higher education curricula should be revised to reflect gender equality. More focused interventions should be given for rural areas. Researchers should also assess other factors (i.e behavioral, environmental, institutional, socioeconomic, and cultural factors) that could affect sexual violence against female university students.

## Supporting information

S1 File PRISMA-P 2009 checklist. (DOC)

S2 File Microsoft excel sheet used for data extraction. (XLSX)

S3 File The electronic search strategy. (DOCX)

## Abbreviations

AOR: Adjusted Odds Ratio
CI: Confidence Interval
EDHS: Ethiopian Demographic and Health Survey
JBI: Joanna Briggs Institute
SDG: Sustainable Development Goal
SNNRR: Southern Nations, Nationalities, and People’s Region
WHO: World Health Organization

## References

[1] UN Women. Elimination and prevention of all forms of violence against women and girls. Commission on the status of women agreed conclusions; 2013.

[2] World Health Organization. Preventing violence against women: a framework for policymakers. World Health Organization; 2019.

[3] Beyene AS, Chojenta C, Roba HS, Melka AS, Loxton D. Gender-based violence among female youths in educational institutions of Sub-Saharan Africa: a systematic review and meta-analysis. Systematic reviews. 2019; 8(1):59 10.1186/s13643-019-0969-9 30803436 PMC6388495

[4] Central Statistical Agency—CSA/Ethiopia, ICF. Ethiopia Demographic and Health Sruvey 2016. Addis Ababa: CSA and ICF; 2017.

[5] Bekele T Deressa W. Prevalence and Experience of Sexual Coercion among Female Students of Ambo University in Ethiopia. Science Journal of Public Health. 2014;2(6): 532–538 10.11648/j.sjph.20140206.16

[6] Henock A, Deyessa N, Abajobir A. Sexual violence and substance use among female students of Mizan-Tepi University, Southwest Ethiopia: A mixed method study. J Womens Health, Issues Care. 2015; 4:4 10.4172/2325-9795.1000194

[7] Bekele T, Kaso M, Gebremariam A, Deressa W. Sexual violence and associated factors among female students of Madawalabu University in Ethiopia. Epidemiology (sunnyvale). 2015;5(2):1000190 10.4172/2161-1165.1000190.

[8] Balcha TT, Lecerof SS, Jeppsson AR. Strategic challenges of PMTCT program implementation in Ethiopia. Journal of the International Association of Physicians in AIDS Care 2011, 10(3):187–192. 10.1177/1545109710369935 21508300

[9] Blum RW, Mmari, Nelson K. Risk and protective factors affecting adolescent reproductive health in developing countries Geneva: Department of Population and Family Health Sciences, Johns HopkinsBloomberg School of Public Health, 2005

[10] Dranzoa C. Sexual Harassment at African Higher Education Institutions. International Higher Education.2018: 94 10.6017/ihe.2018.94.10512

[11] Adinew YM, Hagos MA. Sexual violence against female university students in Ethiopia. BMC international health and human rights. 2017; 17(1):19 10.1186/s12914-017-0127-1 28738807 PMC5525286

[12] Takele A, Setegn T. Sexual coercion and associated factors among female students of Madawalabu University, Southeast Ethiopia. Advances in public health. 2014;2014:417517 10.1155/2014/417517

[13] Arnold D, Gelaye B, Goshu M, Berhane Y, Williams MA. Prevalence and risk factors of gender-based violence among female college students in Awassa, Ethiopia. Violence and Victims. 2008; 23(6):787–800. 10.1891/0886-6708.23.6.787 19069568

[14] Sendo E, Meleku M. Prevalence and factors associated with sexual violence among female students of Hawassa University in Ethiopia. Science Postprint. 2015; 1(2):e00047 10.14340/spp.2015.04A0002

[15] Shimekaw B, Megabiaw B, Alamrew Z. Prevalence and associated factors of sexual violence among private college female students in Bahir Dar city, North Western Ethiopia. 2013;5(6): 1069–1075. 10.4236/health.2013.56143

[16] Benti T, Teferi E. Sexual coercion and associated factors among college female students. J Women’s Health Care.2015;4(4):1000245 10.4172/2167-0420.1000245

[17] Kassa S, Molla A, Cheire N. Sexual Coercion and Determinant Factors among Female Students in Wollo University, Ethiopia; 2019.

[18] Maman S, Campbell J, Sweat MD, Gielen AC. The intersections of HIV and violence: directions for future research and interventions. Social science & medicine. 2000; 50(4):459–478. 10.1016/s0277-9536(99)00270-1 10641800

[19] Andersson N, Cockcroft A, Shea B. Gender-based violence and HIV: relevance for HIV prevention in hyperendemic countries of southern Africa. Aids. 2008; 22:S73–S86. 10.1097/01.aids.0000341778.73038.86 19033757

[20] Campbell JC, Baty M, Ghandour RM, Stockman JK, Francisco L, Wagman J. The intersection of intimate partner violence against women and HIV/AIDS: a review. International Journal of Injury Control and Safety Promotion. 2008; 15(4):221–231. 10.1080/17457300802423224 19051085 PMC3274697

[21] Goodwin MM, Gazmararian JA, Johnson CH, Gilbert BC, Saltzman LE, Group PW. Pregnancy intendedness and physical abuse around the time of pregnancy: findings from the pregnancy risk assessment monitoring system, 1996–1997. Maternal and child health journal. 2000; 4(2):85–92. 10.1023/a:1009566103493 10994576

[22] Pallitto CC, Campbell JC, O’Campo P. Is intimate partner violence associated with unintended pregnancy? A review of the literature. Trauma, Violence, & Abuse. 2005; 6(3):217–235. 10.1177/1524838005277441 16237156

[23] Silverman JG, Gupta J, Decker MR, Kapur N, Raj A. Intimate partner violence and unwanted pregnancy, miscarriage, induced abortion, and stillbirth among a national sample of Bangladeshi women. BJOG: An International Journal of Obstetrics & Gynaecology. 2007; 114(10):1246–1252. 17877676 10.1111/j.1471-0528.2007.01481.x

[24] Henriksen L. Sexual violence, pregnancy and childbirth. Studies investigating the association of experienced sexual violence and outcomes in pregnancy and childbirth; 2015.

[25] Devries KM, Child JC, Bacchus LJ, Mak J, Falder G, Graham K, et al Intimate partner violence victimization and alcohol consumption in women: A systematic review and meta‐analysis. Addiction. 2014; 109(3):379–391. 10.1111/add.12393 24329907

[26] Kennedy AC. Resilience among urban adolescent mothers living with violence: Listening to their stories. Violence Against Women. 2005; 11(12):1490–1514. 10.1177/1077801205280274 16247113

[27] Kennedy AC, Bennett L. Urban adolescent mothers exposed to community, family, and partner violence: Is cumulative violence exposure a barrier to school performance and participation? *Journal of Interpersonal Violence* 2006, 21(6):750–773. 10.1177/0886260506287314 16672740

[28] Vos T, Astbury J, Piers L, Magnus A, Heenan M, Stanley L, et al Measuring the impact of intimate partner violence on the health of women in Victoria, Australia. Bulletin of the World Health Organization. 2006; 84:739–744. 10.2471/blt.06.030411 17128344 PMC2627471

[29] Hyde JS, Mezulis AH, Abramson LY. The ABCs of depression: integrating affective, biological, and cognitive models to explain the emergence of the gender difference in depression. Psychological review. 2008; 115(2):291 10.1037/0033-295X.115.2.291 18426291

[30] Khalifeh H, Dean K. Gender and violence against people with severe mental illness. International review of psychiatry. 2010; 22(5):535–546. 10.3109/09540261.2010.506185 21047165

[31] McPherson MD, Delva J, Cranford JA. A longitudinal investigation of intimate partner violence among mothers with mental illness. Psychiatric Services. 2007; 58(5):675–680. 10.1176/ps.2007.58.5.675 17463349

[32] Ackard DM, Eisenberg ME, Neumark-Sztainer D. Long-term impact of adolescent dating violence on the behavioral and psychological health of male and female youth. The Journal of pediatrics. 2007; 151(5):476–481. 10.1016/j.jpeds.2007.04.034 17961688

[33] Chowdhary N, Patel V. The effect of spousal violence on women’s health: Findings from the Stree Arogya Shodh in Goa, India. Journal of postgraduate medicine. 2008; 54(4):306 10.4103/0022-3859.43514 18953151

[34] Roberts TA, Klein JD, Fisher S. Longitudinal effect of intimate partner abuse on high-risk behavior among adolescents. Archives of pediatrics & adolescent medicine. 2003; 157(9):875–881. 10.1001/archpedi.157.9.875 12963592

[35] Stöckl H, Devries K, Rotstein A, Abrahams N, Campbell J, Watts C, et al The global prevalence of intimate partner homicide: a systematic review. The Lancet. 2013; 382(9895):859–865. 10.1016/S0140-6736(13)61030-2 23791474

[36] García-Moreno C, Jansen H, Ellsberg M, Heise L, Watts C. WHO multi-country study on women’s health and domestic violence against women. Geneva: World Health Organization. 2005; 204:1–18.

[37] Pietilä H, Peoc’h B. The unfinished story of women and the United Nations: UN; 2007.

[38] Assembly G: Resolution adopted by the General Assembly on 19 September 2016. In.: A/RES/71/1, 3 October 2016 (The New York Declaration); 2010.

[39] United Nations General Assembly. Resolution adopted by the general assembly on 25 September 2015: transforming our world: the 2030 agendafor sustainable development. New York: United Nations; 2015.

[40] The Federal Democratic Republic of Ethiopia. The Criminal Code of the Federal Democratic Republic of Ethiopia Proclamation No.414/2004

[41] The Federal Democratic Republic of Ethiopia. The Revised Family Code Proclamation No. 213/2000: Federal Negarit Gazetta Extra Ordinary Issue No. 1/2000

[42] Liberati A, Altman DG, Tetzlaff J, Mulrow C, Gøtzsche PC, Ioannidis JP, et al The PRISMA statement for reporting systematic reviews and meta-analyses of studies that evaluate health care interventions: explanation and elaboration. Annals of internal medicine. 2009; 151(4):W-65–W-94. 19622512 10.7326/0003-4819-151-4-200908180-00136

[43] WHO. Violence against women: intimate partner and sexual violence against women: evidence brief. In: Violence against women: intimate partner and sexual violence against women: evidence brief. edn.; 2019.

[44] The Joanna Briggs Institute. Joanna Briggs Institute Reviewers’ Manual: 2014 edition The Joanna Briggs Institute 2014.

[45] Higgins JP, Thompson SG, Deeks JJ, Altman DG. Measuring inconsistency in meta-analyses. Bmj. 2003; 327(7414):557–560. 10.1136/bmj.327.7414.557 12958120 PMC192859

[46] Begg CB, Mazumdar M. Operating characteristics of a rank correlation test for publication bias. Biometrics. 1994; 1088–1101. 7786990

[47] Egger M, Smith GD, Schneider M, Minder C. Bias in meta-analysis detected by a simple, graphical test. Bmj. 1997, 315(7109):629–634. 10.1136/bmj.315.7109.629 9310563 PMC2127453

[48] Ji K, Finkelhor D, Dunne M. Child sexual abuse in China: A meta-analysis of 27 studies. Child Abuse & Neglect. 2013; 37(9):613–622. 10.1016/j.chiabu.2013.03.008 23643201

[49] Stoltenborgh M, Van Ijzendoorn MH, Euser EM, Bakermans-Kranenburg MJ. A global perspective on child sexual abuse: meta-analysis of prevalence around the world. Child maltreatment. 2011; 16(2):79–101. 10.1177/1077559511403920 21511741

[50] Pereda N, Guilera G, Forns M, Gómez-Benito J. The prevalence of child sexual abuse in community and student samples: A meta-analysis. Clinical psychology review. 2009; 29(4):328–338. 10.1016/j.cpr.2009.02.007 19371992

[51] Barth J, Bermetz L, Heim E, Trelle S, Tonia T. The current prevalence of child sexual abuse worldwide: a systematic review and meta-analysis. International journal of public health. 2013; 58(3):469–483. 10.1007/s00038-012-0426-1 23178922

[52] Kearns MC, D’inverno AS, Reidy DE. The association between gender inequality and sexual violence in the US. American journal of preventive medicine 2020, 58(1):12–20. 31761512 10.1016/j.amepre.2019.08.035 PMC7810166

[53] Butchart A, Garcia-Moreno C, Mikton C. Preventing intimate partner and sexual violence against women: taking action and generating evidence; 2010. 10.1136/ip.2010.029629 20921563

[54] Ayalew S. The Security and Human Rights Dilemma: An Inquiry into US-Ethiopia Diplomatic Relations 1991–2012. Nw UJ Int’l Hum Rt. 2018; 16:65 https://scholarlycommons.law.northwestern.edu/njihr/vol16/iss1/4

[55] UN General Assembly. In-depth study on all forms of violence against women: report of the Secretary-General; 2006. https://www.refworld.org/docid/484e58702.html

[56] Kedir A, Admasachew L. Violence against women in Ethiopia. *Gender*, *place & culture* 2010, 17(4):437–452. 10.1080/0966369X.2010.485832

[57] Klostermann KC, Fals-Stewart W. Intimate partner violence and alcohol use: Exploring the role of drinking in partner violence and its implications for intervention. Aggression and violent behavior. 2006; 11(6):587–597.

[58] Lane SD, Cherek DR, Pietras CJ, Tcheremissine OV. Alcohol effects on human risk taking. Psychopharmacology. 2004; 172(1):68–77. 10.1007/s00213-003-1628-2 14647967

[59] Tumwesigye NM, Kasirye R, Nansubuga E. Is social interaction associated with alcohol consumption in Uganda? Drug and alcohol dependence. 2009; 103(1–2):9–15. 10.1016/j.drugalcdep.2009.01.016 19406589

[60] WHO. Global and regional estimates of violence against women: prevalence and health effects of intimate partner violence and non-partner sexual violence: World Health Organization; 2013.

