# Supplementary material for: Predictors of sexual violence among female students in higher education institutions in Ethiopia: A systematic review and meta-analysis
Source: PLoS One. 2021 Feb 19;16(2):e0247386. doi: 10.1371/journal.pone.0247386 (PMC7894927; doi:10.1371/journal.pone.0247386)
Supplement: S3 File — (DOCX) [file pone.0247386.s003.docx]

((((((((((((((((((((((((((prevalence) OR (proportion)) OR (incidence)) OR (magnitude)) AND ("sexual violence")) OR ("sexual abuse")) OR ("sexual coercion")) OR ("sexual harassment")) OR ("sexual assault")) OR (violence)) OR (rape)) OR ("gender-based violence")) OR (assault)) AND (factors)) OR (determinants)) OR (predictors)) OR ("factors associated")) OR ("associated factors")) OR ("risk factors")) AND (University)) AND (College)) OR ("Higher education institutions")) OR (campus)) AND (students)) OR ("undergraduate students")) AND (Female)) AND (Ethiopia)
